# Factors influencing decisions people with motor neuron disease make about gastrostomy placement and ventilation: A qualitative evidence synthesis

**DOI:** 10.1111/hex.13786

**Published:** 2023-05-31

**Authors:** Sean White, Alicia O'Cathain, Vanessa Halliday, Liz Croot, Christopher J. McDermott

**Affiliations:** ^1^ Neurosciences Department, Faculty of Medicine Dentistry and Health The University of Sheffield Sheffield UK; ^2^ School of Health and Related Research (ScHARR) The University of Sheffield Sheffield UK

**Keywords:** amyotrophic lateral sclerosis, decision‐making, gastrostomy, motor neuron disease, qualitative, ventilation

## Abstract

**Background:**

People with motor neuron disease (pwMND) are routinely offered gastrostomy feeding tube placement and (non‐invasive and invasive) ventilation to manage the functional decline associated with the disease. This study aimed to synthesise the findings from the qualitative literature to understand how individual, clinical team and organisational factors influence pwMND decisions about these interventions.

**Methods:**

The study design was guided by the enhancing transparency in reporting the synthesis of qualitative research (ENTREC) statement. The search of five bibliography databases and an extensive supplementary search strategy identified 27 papers that included qualitative accounts of pwMND, caregivers and healthcare professionals' (HCPs) experiences of making decisions about gastrostomy and ventilation. The findings from each study were included in a thematic synthesis.

**Findings:**

Making decisions about interventions is an emotional rather than simply a functional issue for pwMND. The interventions can signal an end to normality, and increasing dependence, where pwMND consider the balance between quality of life and extending survival. Interactions with multiple HCPs and caregivers can influence the process of decision‐making and the decisions made. These interactions contribute to the autonomy pwMND are able to exert during decision‐making. HCPs can both promote and threaten pwMND perceived agency over decisions through how they approach discussions about these interventions. Though there is uncertainty over the timing of interventions, pwMND who agree to interventions report reaching a tipping point where they accept the need for change.

**Conclusion:**

Discussion of gastrostomy and ventilation options generate an emotional response in pwMND. Decisions are the consequence of interactions with multiple external agents, including HCPs treading a complex ethical path when trying to improve health outcomes while respecting pwMND right to autonomy. Future decision support interventions that address the emotional response and seek to support autonomy have the potential to enable pwMND to make informed and timely decisions about gastrostomy placement and ventilation.

**Patient or Public Contribution:**

The lead author collaborated with several patient and participant involvement (PPI) groups with regards to the conceptualisation and design of this project. Decisions that have been influenced by discussions with multiple PPI panels include widening the scope of decisions about ventilation in addition to gastrostomy placement and the perceptions of all stakeholders involved (i.e., pwMND, caregivers and HCPs).

## 1 INTRODUCTION

Motor neuron disease (MND), also known as amyotrophic lateral sclerosis (ALS), is a progressive neurological condition with a global incidence of 1.59 per 100,000 person‐years and prevalence of 4.42 per 100,000 population.^1^ MND is associated with a 2–5 year survival after symptom onset with a lack of therapeutic options that can delay disease progression.^2^, ^3^ The clinical management of MND focuses on compensating for the progressive loss of vital physiological functions including respiratory failure and swallowing difficulties.^4^

People with MND (pwMND) are routinely presented with options to start interventions aimed at improving quality of life and/or extending survival.^3^ Interventions offered include gastrostomy placement, providing an alternative route for administering nutrition and hydration, and ventilation (non‐invasive and invasive) for respiratory support.^5^, ^6^ Clinical guidance recommends discussing such interventions early in the disease course and the timely initiation of interventions to reduce procedural complications and promote better outcomes.^7^, ^8^, ^9^ Though these interventions have the potential to extend survival,^10^, ^11^, ^12^, ^13^ they can also address the negative impact that functional losses have on pwMND quality of life.^3^ In addition to the proposed benefits, the risks and burdens should also be considered during decision‐making.^14^, ^15^

pwMND make decisions in the context of an often rapidly progressive disease with no hope of a cure. The risks and benefits associated with interventions are continually changing as the health threats presented by symptom progression escalate. King et al.^16^ developed a cyclical model of decision‐making describing how pwMND repeatedly respond and adapt to the relentless step‐changes in their condition. There are a range of contextual and relational factors that influence how, what and when, pwMND make decisions about their care.^17^, ^18^ Decisions are the result of interactions with a range of external agents including caregivers and the multidisciplinary team (MDT) supporting them.^18^, ^19^, ^20^ In addition, cognitive and behavioural changes prevalent in MND impact on the abilities of the individual to make decisions and the support they need to do so, adding to the complex context in which pwMND make decisions about their care.^21^, ^22^ There are a growing number of qualitative studies that have captured the perspectives of pwMND, caregivers and healthcare professionals' (HCPs) during decision‐making about gastrostomy and ventilation. This synthesis and further conceptualisation of decision‐making about these interventions in MND care from multiple perspectives, will inform the future development of contextually sensitive decision support strategies.

The aim of this qualitative evidence synthesis is to understand how individual, clinical team and organisational factors influence the decisions that pwMND make about gastrostomy and ventilation (for the purpose of this paper ‘gastrostomy placement and ventilation [non‐invasive and invasive]’ will be referred to as ‘interventions’ from this point onwards).

## 2 METHODS

### 2.1 Design

A qualitative evidence synthesis was selected to systematically identify and synthesise the findings from the published qualitative literature, to gain a broad and rich understanding of the factors that influence decision‐making about interventions in MND care.^23^ The study design was guided by the enhancing transparency in reporting the synthesis of qualitative research (ENTREC) statement (Supporting Information: Appendix SA).^24^ The protocol was registered on PROSPERO on October 14, 2021 (CRD42021283314).

### 2.2 Search strategy

The search strategy was informed by a scoping search and in consultation with subject experts, academic information specialists, patient and public involvement groups and the research team. All searches took place between September and October 2021. Free text terms and subject headings related to the concepts ‘motor neuron disease’, ‘decision‐making’, ‘gastrostomy OR ventilation’ and ‘qualitative study’ were combined using the Boolean term ‘AND’. The search strategies were adapted for searches on five bibliographic databases: Medline, Embase, CINAHL, PsycINFO and Cochrane Library database (see Supporting Information: Appendix SB for the full Medline search terms). An extensive supplementary search strategy was conducted to identify any references not captured by the bibliographic database searches (Supporting Information: Appendix SC). The reference lists of included studies, relevant guidelines and reviews, and the previous 3 years' contents of the ‘Amyotrophic Lateral sclerosis and Frontotemporal Degeneration’ journal and the International Symposium on ALS/MND conference proceedings were hand searched. A forward citation check of all the studies included in the review was performed using the Web of Science database. The authors of the included studies and selected topic matter experts were contacted to identify further studies that may meet the inclusion criteria.

### 2.3 Inclusion criteria

Studies were included if they contained qualitative accounts of pwMND, caregivers or HCPs making decisions about interventions. Caregivers were defined as being unpaid people who support pwMND. Only studies in the English language were included and no date restrictions were set.

### 2.4 Study screening and selection

**Figure 1:**
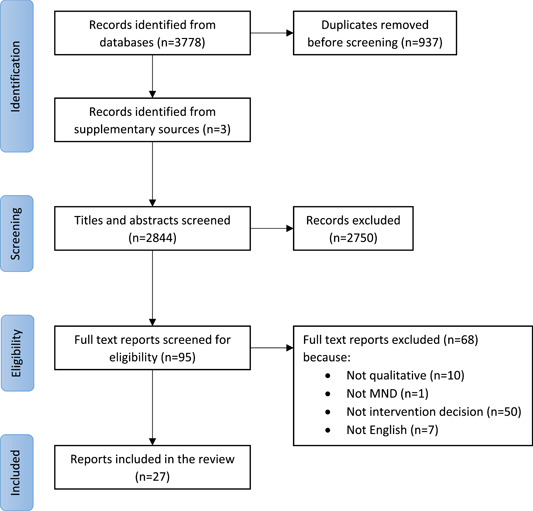
Results of the study screening and selection process.

A total of 3781 references identified by the database and supplementary searches were imported into EndNote 20 reference manager. Following the removal of 937 duplicates, the title and abstracts of 2844 references were screened for inclusion by S.W. A total of 95 full‐text papers were screened for inclusion in the review by S.W. L.C. screened 10% of the references at both the title/abstract and full‐text screening stages. Any disagreements were discussed and resolved between S.W. and L.C., with further discussions with C.M., A.C. and V.H. when required. Finally, 26 papers met the inclusion criteria to be included in the review. The supplementary searches identified one further paper for inclusion.^25^ See Figure 1 for the full results of the study screening and selection process.

### 2.5 Quality assessment

The Critical Appraisal Skills Programme (CASP) qualitative checklist was used to assess the quality and rigour of the included papers (Table 1).^26^ The quality assessment did not guide study selection due to the documented concerns relating to the consistency and subjectivity of such appraisals.^52^, ^53^, ^54^ Two studies scored poorly across most domains of the CASP checklist. Leslie^38^ did not include any methods, analysis or discussion, but the transcripts from the interviews provided interesting insights that were relevant to the aims of this review. Versalovic and Klein^49^ paper lacked information about the study design and analysis, but the richness of the accounts addressed the review aim. Only six studies discussed the researchers' relationship (i.e., reflexivity) with the participants.^27^, ^36^, ^37^, ^42^, ^45^, ^51^

**Table 1:** Quality assessment of the included papers using the CASP checklist.^26^

Study year	1	2	3	4	5	6	7	8	9	10
Ando et al. (2015)^27^	Y	Y	Y	Y	Y	Y	Y	Y	Y	Y
Chapman et al. (2021)^25^	Y	Y	Y	Y	Y	N	Y	Y	Y	Y
Clarke et al. (2018)^28^	Y	Y	Y	Y	Y	N	Y	Y	Y	C
Foley et al. (2014)^29^	Y	Y	Y	Y	Y	N	Y	Y	Y	Y
Foley et al. (2014)^30^	Y	Y	Y	Y	Y	N	Y	Y	Y	Y
Foley et al. (2016)^31^	Y	Y	Y	Y	Y	N	Y	Y	Y	Y
Gottberg et al. (2021)^32^	Y	Y	Y	Y	Y	N	Y	Y	Y	Y
Greenaway et al. (2015)^33^	Y	Y	Y	Y	Y	N	Y	Y	Y	Y
Hirano and Yamazaki (2010)^34^	Y	Y	Y	Y	Y	N	Y	Y	Y	Y
Hodgins et al. 2020^35^	Y	Y	Y	Y	Y	N	Y	Y	Y	Y
Hogden et al. (2012)^17^	Y	Y	Y	Y	Y	N	Y	Y	Y	Y
Labra et al. (2020)^36^	Y	Y	Y	Y	Y	Y	Y	Y	Y	Y
Lemoignan and Ells (2010)^37^	Y	Y	Y	Y	Y	Y	Y	Y	Y	Y
Leslie (2008)^38^	N	Y	N	N	N	N	N	N	N	C
Martin et al. (2016)^39^	Y	Y	Y	Y	Y	N	Y	Y	Y	Y
Murray et al. (2016)^40^	Y	Y	Y	Y	Y	N	C	Y	Y	Y
Nolan et al. (2008)^41^	Y	Y	Y	Y	Y	N	C	Y	Y	Y
Paynter et al. (2020)^42^	Y	Y	Y	Y	Y	Y	Y	Y	Y	Y
Pols and Limburg (2016)^43^	Y	Y	Y	Y	Y	N	C	N	Y	Y
Sakellariou (2016)^44^	Y	Y	Y	C	Y	N	C	Y	Y	Y
Seeber et al (2019)^45^	Y	Y	Y	Y	Y	Y	Y	Y	Y	Y
Stavroulakis et al (2014)^46^	Y	Y	Y	Y	Y	N	C	Y	Y	Y
Sundling et al. (2009)^47^	Y	Y	C	Y	Y	C	Y	Y	Y	Y
Veronese et al. (2014)^48^	Y	Y	Y	C	C	N	Y	Y	Y	Y
Versalovic and Klein (2020)^49^	C	C	C	N	N	N	N	N	C	Y
Whitehead et al. (2011)^50^	Y	Y	Y	Y	Y	N	Y	Y	Y	Y
Young et al. (1994)^51^	Y	Y	C	Y	Y	Y	N	C	Y	Y

*Note*: Results of the quality assessment using the CASP qualitative checklist.^26^ Criteria labels: 1. Was there a clear statement of the aims of the research? 2. Is a qualitative methodology appropriate? 3. Was the research design appropriate to address the aims of the research? 4. Was the recruitment strategy appropriate to the aims of the research? 5. Was the data collected in a way that addressed the research issue? 6. Has the relationship between the researcher and participants been adequately considered? 7. Have the ethical issues been taken into consideration? 8. Was the data analysis sufficiently rigorous? 9. Is there a clear statement of findings? 10. How valuable is the research? (note: Question 10 of the CASP tool does not seek a yes/no/can't tell answer; yes has been selected when the author does address the hints provided in the tool).

Abbreviations: C, can't tell; CASP, Critical Appraisal Skills Programme; N, no; Y, yes.

### 2.6 Study characteristics

This review includes a synthesis of the findings from 25 studies reported in 27 papers, (three papers included the qualitative analysis of the same participant cohort from one study^29^, ^30^, ^31^). Of the 430 participants in the 25 studies, 241 were pwMND (where stated: 118 male, 75 female), 103 were caregivers (where stated: 35 male, 52 female) and 85 were HCPs (genders not stated). One study did not include any information about participants.^49^ All the studies used interviews, with two studies also including observations^43^, ^45^ and another group interviews.^17^ The papers included qualitative accounts of making decisions about gastrostomy alone (*N* = 12), ventilation (non‐invasive and invasive) alone (*N* = 8) and both gastrostomy and ventilation (*N* = 7). The studies were conducted in a number of countries including: United Kingdom (*N* = 8); Australia (*N* = 5); The Netherlands (*N* = 2); Sweden (*n* = 2); United States of America (*N* = 2); Canada (*N* = 2); Ireland (*N* = 1); Japan (*N* = 1); Italy (*N* = 1); not available (*N* = 1). See Table 2 for the study characteristics.

**Table 2:** Characteristics of the papers included in the review.

Author (year)	Country	Study aim	Perspective	Intervention	Study design/methodology	Sample size: total and per participant type; (gender)
Ando et al. (2015)^27^	UK	Explore why pwMND declined or withdrew NIV, to understand patient experience of being offered NIV.	Patients	NIV	Semi‐structured interviews	9 (M = 7; F = 2)
Chapman et al. (2021)^25^	Australia	Explore communication between pwMND and their clinicians about NIV and gastrostomy.	Patients, HCP, caregivers	Gastrostomy and NIV	Semi‐structured interviews	26; pwMND = 1; caregivers = 6; HCP = 19; (gender N/A)
Clarke et al. (2018)^28^	UK	What are the experiences and views of pwMND and their families on decision‐making concerning their care, with a focus on problems with eating and drinking.	Patients, caregivers	Gastrostomy	Qualitative interviews	7; 4 pwMND (gender N/A), 3 caregivers (M = 1; F = 2)
Foley et al. (2014)^29^	Ireland	Identify processes that underpin how and why people with ALS engage with healthcare services.	Patients	Gastrostomy	Qualitative interviews	34 (M = 17; F = 17)
Foley et al. (2014)^30^	Ireland	Identify key psychosocial processes that underpin how people with MND engage with their services.	Patients	Gastrostomy and NIV	Qualitative interviews	34 (M = 17; F = 17)
Foley et al. (2016)^31^	Ireland	Exploring pwMND experiences of receiving care from family members and formal service providers.	Patients	NIV	Qualitative interviews	34 (M = 17; F = 17)
Gottberg et al. (2021)^32^	Sweden	Investigate the experience of caregivers for pwMND on invasive ventilation via tracheostomy.	Caregivers	Mechanical ventilation via tracheostomy	Semi‐structured interviews	8 (M = 2; F = 6)
Greenaway et al. (2015)^33^	UK	To identify factors that affect pwMND accepting or declining NIV and gastrostomy.	Patients	Gastrostomy and NIV	Semi‐structured interviews	21 (M = 13; F = 8)
Hirano and Yamazaki (2010)^34^	Japan	Investigating factors affect pwMND decision‐making about invasive mechanical ventilation.	Patients	Invasive mechanical ventilation	Semi‐structured interviews	50 (M = 34; F = 16)
Hodgins et al. (2020)^35^	UK	Evaluate the impact of the Edinburgh Cognitive and Behavioural ALS Screen on pwMND, caregivers and HCPs.	Patients, caregivers and HCPs	Gastrostomy	Semi‐structured interviews	21 (Gender N/A)
Hogden et al. (2012)^17^	Australia	To explore patient decision‐making for symptom management from the experience of health professionals and to identify factors influencing decision‐making in specialised multidisciplinary ALS care.	HCPs	Gastrostomy and ventilation	In‐depth interviews and group interviews	32 (Gender N/A)
Labra et al. (2020)^36^	Australia	What are the factors that impact on pwMND uptaking gastrostomy.	Patients	Gastrostomy	Mixed methods including standardised assessments, nutrition survey and semi‐structured interview	10 (M = 6; F = 4)
Lemoignan and Ells (2010)^37^	Canada	To explore the experience of decision‐making about assisted ventilation for pwMND.	Patients	NIV and mechanical ventilation	Semi‐structured interviews	9 (M = 6; F = 3)
Leslie (2008)^38^	USA	To explore patients' thoughts on information, values, outside pressure, support and their reflections back on the process of making decisions about PEG.	Patients	Gastrostomy	Semi‐structured interview	2 (F = 2)
Martin et al. (2016)^39^	UK	To investigate factors affecting decision‐making about gastrostomy and NIV by people with ALS from the viewpoint of the HCPs supporting them through their decision‐making.	HCPs	Gastrostomy and NIV	In‐depth qualitative interviews	19 (Gender N/A)
Murray et al. (2016)^40^	Australia	To elicit the experiences of bereaved caregivers of MND patients who had or had not completed the letter of future care.	Caregivers	Gastrostomy	Semi‐structured interviews	18 (M = 5; F = 13)
Nolan et al. (2008)^41^	USA	Compare the preferences of patients with ALS for involving family in healthcare decisions at the end of life with the actual involvement reported by the family after death.	Caregivers	Gastrostomy	In‐depth qualitative interviews	16 (M = 8; F = 8)
Paynter et al. (2020)^42^	Australia	To explore involvement and engagement in decision‐making, and how this was affected by communication or cognitive impairments.	Patients and caregivers	Gastrostomy and NIV	Semi‐structured interviews	35; 19 pwMND (M = 10; F = 9), 15 caregivers (M = 5; F = 10)
Pols and Limburg (2016)^43^	The Netherlands	To learn more about what the meaning of the term quality of life means when it is studied in daily life, in reference to gastrostomy feeding in ALS care.	Patients, caregivers and HCPs	Gastrostomy	Nonparticipant observation (*N* = 28) and semi‐structured interviews (*N* = 11)	39; 11 pwMND interviewed (gender N/A); 28 pwMND observed (gender N/A)
Sakellariou (2016)^44^	UK	How do people involved in relationships of care enact subjectivity.	Patients and caregivers	Gastrostomy	Interviews	2; 1 pwMND (F = 1), 1 caregiver (M = 1)
Seeber et al. (2019)^45^	The Netherlands	Evaluate timing and content of discussions about treatments and end‐of‐life care.	Patients	Gastrostomy and NIV	Nonparticipant observation and in‐depth interviews	21; (M13; F = 8)
Stavroulakis et al. (2014)^46^	UK	To explore the decision‐making process in relation to timing of gastrostomy insertion from the perspective of the patients and their informal carers.	Patients and caregivers	Gastrostomy	Semi‐structured interviews	18; 10 pwMND (M = 7; F = 3), 8 caregivers (gender N/A)
Sundling et al. (2009)^47^	Sweden	To describe the experiences of patients with ALS as well as their caregivers, of noninvasive positive pressure ventilation.	Patient and caregiver	NIV	Interviews	15; 7 pwMND (M = 5; F = 2), 8 caregivers (M = 2; F = 6)
Veronese et al. (2014)^48^	Italy	To identify how the decision of a tracheostomy was taken by the patients.	Caregivers	Tracheostomy	Semi‐structured interviews	19; spouses = 11, children = 7, paid carer = 1 (gender N/A)
Versalovic and Klein (2020)^49^	N/A	To elucidate the ways patients make sense of who they are and who they will be at later stages of illness through their conversations with and considerations of the others around them.	Patients	Gastrostomy	Interviews	NK
Whitehead et al. (2011)^50^	UK	To explore the experiences of people with motor neurone disease, current and bereaved carers in the final stages of the disease and bereavement period.	Caregivers	NIV	Narrative interviews	18 (M = 11; F = 7)
Young et al. (1994)^51^	Canada	To identify the factors involved when pwMND are making a decision to start mechanical ventilation.	Patients	Mechanical ventilation	Semi‐structured interviews	13 (Gender N/A)

Abbreviations: ALS, amyotrophic lateral sclerosis; F, female; HCP, healthcare professional; M, male; MND, motor neuron disease; N/A, not available; NIV, noninvasive ventilation; pwMND, person with MND.

### 2.7 Data synthesis

A scoping search of the current evidence and the resources available informed the decision to perform a thematic synthesis.^55^ Thematic synthesis allows for the generation of analytical findings that conceptualise how decisions are made in the context of MND care. The methods described by Thomas and Harden,^56^ were followed including: (1) line‐by‐line coding of text; (2) developing descriptive themes; (3) developing overarching analytical themes.

Papers were imported into QSR NVivo (Version 13) for coding and analysis by S.W. Only the text present in the findings section of the papers were extracted for the analysis.^56^, ^57^ All of the papers were read and re‐read to increase familiarity with the data before coding. Codes were organised into descriptive themes that remained close to the original data. The final phase involved moving beyond the categorisation of descriptive themes and onto the development of analytical themes.

## 3 FINDINGS

**Figure 2:**
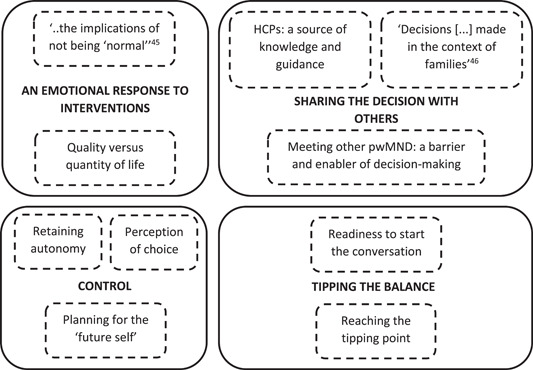
A summary of the four analytical themes (in capitals and bold) and subthemes (boxes with dashed lines) developed as a result of the thematic synthesis.

Four analytical themes were developed describing the factors impacting on pwMND decisions about gastrostomy and ventilation: ‘An emotional response to interventions’; ‘Sharing the decision with others’; ‘Control’; and ‘Tipping the balance’ (Figure 2). The analytical themes describe the intrinsic, contextual and relational factors that influence how and when pwMND make decisions about interventions. Verbatim quotes, punctuated with single punctuation marks, taken from the original papers are included to support the analytical findings, with the source of quotes indicated by pwMND (P), caregiver (C), HCP (H) or researcher (R). Table 3 describes the contribution each paper made to the analytical themes.

**Table 3:** The contribution made by each paper to the themes.

Study	An emotional response to interventions		Sharing the decision with others			Control			Tipping the balance	
	‘…the implications of not being “normal”’^46^	Quality versus quantity of life	HCPs: A source of knowledge and guidance	‘Decisions […] made in the context of families’^39^	Meeting other pwMND: A barrier and enabler of decision‐making	Retaining autonomy over decisions	When the perception of choice runs out	Planning for the ‘future self’	Readiness to start the conversation	Reaching the tipping point
Ando et al. (2015)^27^	✔	✔	✔		✔	✔				✔
Chapman et al. (2021)^25^			✔					✔		✔
Clarke et al. (2018)^28^				✔				✔		
Foley et al. (2014)^29^						✔		✔	✔	✔
Foley et al. (2014)^30^		✔				✔		✔		
Foley et al. (2016)^31^		✔		✔						
Gottberg et al. (2021)^32^				✔		✔	✔			✔
Greenaway et al. (2015)^33^	✔	✔	✔	✔		✔	✔	✔	✔	✔
Hirano and Yamazaki (2010)^34^	✔	✔	✔		✔		✔		✔	✔
Hodgins et al. 2020^35^						✔		✔		
Hogden et al. (2012)^17^			✔						✔	
Labra et al. (2020)^36^	✔	✔						✔		✔
Lemoignan and Ells (2010)^37^	✔	✔	✔	✔		✔	✔	✔	✔	✔
Leslie (2008)^38^	✔	✔	✔	✔		✔			✔	✔
Martin et al. (2016)^39^	✔	✔	✔	✔	✔	✔	✔	✔	✔	✔
Murray et al. (2016)^40^	✔				✔	✔		✔		
Nolan et al. (2008)^41^								✔		
Paynter et al. (2020)^42^	✔	✔	✔				✔		✔	✔
Pols and Limburg (2016)^43^	✔	✔	✔					✔	✔	✔
Sakellariou (2016)^44^	✔					✔	✔			
Seeber et al. (2019)^45^			✔						✔	✔
Stavroulakis et al. (2014)^46^	✔		✔		✔	✔	✔	✔	✔	✔
Sundling et al. (2009)^47^						✔				
Veronese et al. (2014)^48^		✔				✔	✔			✔
Versalovic and Klein (2020)^49^								✔		
Whitehead et al. (2011)^50^	✔			✔	✔	✔	✔	✔		
Young et al. (1994)^51^	✔	✔				✔		✔		

Abbreviations: HCP, healthcare professional; pwMND, person with MND.

### 3.1 An emotional response to interventions

The prospect of starting an intervention represented a threat to pwMND sense of normality including the implications for their level of independence, freedom, and reliance on others. Additionally, pwMND perceptions were influenced by how they value an interventions' potential to prolong life and the quality of that remaining life.

#### 3.1.1 ‘…The implications of not being “normal”’^46^

pwMND deliberations about whether to commence interventions extend beyond the clinical problems they are proposed to ameliorate. Decision‐making is influenced by a range of emotional responses with regard to what the interventions signify and how daily life with them will compare to the current status quo.

Discussions about interventions acted as an acute reminder for some pwMND of disease progression.^27^, ^37^, ^39^, ^42^, ^43^, ^44^ Acceptance of the need for interventions was associated with giving in to the disease^39^ and signified a stage of the disease associated with a poor quality of life or nearing the end of life.^27^, ^37^, ^39^

(H) ‘*…A very obvious sign of being ill…a very concrete representation that he's seriously ill.’* ^39^

An emotional response was generated when learning about the procedures involved,^33^, ^38^, ^39^, ^42^, ^43^, ^46^ a requirement to come into hospital^27^, ^36^, ^39^ and threats to pwMND physical integrity.^27^, ^33^, ^38^, ^40^, ^43^ For some pwMND their response to the prospect of interventions was influenced by previous negative healthcare experiences.^27^, ^33^, ^39^

Additionally, pwMND were concerned about how interventions may impact on their current sense of normality. pwMND reported contrasting views about how an intervention may enable or hinder their ability to engage in social activities^27^, ^38^, ^39^, ^51^ or alter their reliance on care from others.^34^, ^36^, ^37^, ^39^, ^50^, ^51^ While some pwMND were able to visualise the gains interventions could facilitate in terms of freeing time or reducing burden on caregivers,^36^, ^39^, ^43^, ^46^ most studies reported how they represented changes to ‘normal life’ that they were unwilling to accept easily.

(R) ‘*All participants talked about the affect of QOL (including the ability to communicate, eat, move around, and be surrounded by loved ones) on decisions about assisted ventilation.’* ^37^

#### 3.1.2 Quality versus quantity of life

Existential views on the potential for interventions to prolong life were integrated into pwMND decision‐making. A number of studies reported pwMND preference to prioritise quality of life over prolonging a life that lacked value to them.^27^, ^30^, ^33^, ^36^, ^37^, ^39^ Knowledge about the terminal phases of MND could even bring about a preference to actively shorten life through refusing an intervention.^36^, ^51^

(R) ‘*Most participants suggested that losing independence rendered life less meaningful and that life‐sustaining interventions had the potential to prolong ‘suffering’*.’^30^

Reassurance from others and an overriding belief that quality of life will be maintained or improved, promoted a positive perception of interventions.^33^, ^34^, ^36^, ^37^, ^38^, ^39^, ^42^, ^43^ While some HCPs discussed interventions in relation to the impact on quality of life,^39^ there were also examples of HCPs goals focusing on clinical outcomes.

(R) ‘*…the sensual qualities of the body surprised the gastroenterologist, who saw maintaining physical fitness and longer survival as the ultimate goals, and the feeding tube as a means towards this end*…’^43^

pwMND reported considering how any extension to their lives may impact on their caregivers. Interventions were perceived to lengthen the time they are a burden on caregivers^36^ or, in contrast, facilitated them to be with or support family for longer.^33^, ^34^, ^39^, ^43^ For some pwMND extending life was an overriding goal, particularly when faced with a direct threat to life such as in crisis situations.^30^, ^33^, ^37^, ^39^, ^42^, ^48^, ^51^

(P) ‘*Any living person's will to survive is primordial and outweighs many other concerns or reasons.’* ^37^

pwMND perceptions of interventions are both intrinsically generated and the consequence of interactions with multiple external agents. The following theme captures how HCPs, caregivers and other pwMND can influence the decision‐making process.

### 3.2 Sharing the decision with others

pwMND decision‐making involves interactions with a range of external agents. HCPs share information and attempt to guide pwMND through the decision; caregivers share some of the burden of decision‐making with pwMND; and knowledge of other pwMND can help clarify or challenge perceptions and values.

#### 3.2.1 HCPs: A source of knowledge and guidance

The style, amount, and pace of information shared by HCPs influenced pwMND experience of the decision‐making process. HCPs focusing on delivering comprehensive information about interventions, may prevent pwMND from having the opportunity to understand and explore the issues most important to them.^25^, ^33^ Some HCPs reported following a structured approach to supporting pwMND decision‐making through seeking permission to initiate discussions, presenting options available, exploring preferences and distributing discussions over multiple interactions.^37^, ^38^, ^43^ HCPs using a respectful, personable communication style including empathy and reassurance was valued by pwMND.^33^

(C) ‘*So, you know, some health professionals—I think periods of silence to listen to the client are needed without the health professional thinking, “I've got to tell them X, Y, Z…”*’^25^

HCPs were a trusted source of information and pwMND expected them to use their expertise to guide decision‐making.^33^, ^34^, ^38^, ^39^, ^46^ However, credibility of healthcare services was threatened by a perceived lack of HCP knowledge,^33^ poor experiences of healthcare services,^27^ or a lack of clear guidance.^33^ A breakdown in trust in HCPs or healthcare services may lead to disengagement of pwMND from the decision‐making process.

pwMND reported having multiple interactions with different HCP roles when considering interventions.^25^, ^33^, ^34^, ^38^, ^39^, ^45^, ^46^ HCPs expressed challenges with the MDT coordinating decision support including pwMND being given contradictory information from different team members^25^, ^33^, ^39^ and poor communication between services.^17^, ^25^, ^42^

(H) ‘*I think my…fear is you can get so many teams of people involved. […] the possibilities of confusion and misinformation are enormous…I was going to say warfare [can occur].’* ^25^

#### 3.2.2 ‘Decisions […] made in the context of families’^39^

Caregivers were reported to play a supportive role, through seeking information, expressing opinions and deliberating about options.^31^, ^33^, ^37^, ^38^ Through these interactions caregivers ‘influenced not only the decision but also the process of decision‐making’.^39^ Caregivers required information early in the disease process to facilitate their role supporting pwMND decision‐making.^28^, ^37^ While caregivers recognised the pwMND right to self‐determination, their contributions to the decision‐making process were not always described as being neutral and were informed by their own preferences and acceptance of the diagnosis.^32^, ^33^, ^39^ pwMND preferences for or against interventions were challenged when caregivers preferences were not aligned with those of the pwMND.^31^, ^33^, ^39^, ^50^

(R) ‘*Some patients experienced their families more negatively, with the family members' emotional needs taking precedence over those of the patient.’* ^33^

#### 3.2.3 Meeting other pwMND: A barrier and enabler of decision‐making

Understanding experiences of other pwMND facilitated some pwMND to clarify their own preferences, including learning about pwMND reflections of delaying decision‐making, reassurance that they could adapt to life with an intervention and informing values with regard to prolonging life.^27^, ^34^, ^39^, ^40^, ^46^

(P) ‘*The decisive factor was meeting ALS patients already using it. These people with the same disease had a positive outlook on life, and this gave me the will to live as well. My uncertainty disappeared.’* ^27^

However, others reported meeting other pwMND would have been a barrier to decision‐making, reminding them about the future symptom burden they would be living with and may wish to avoid.^34^, ^50^

The involvement of multiple external agents raises the question of who is responsible for making decisions and how interactions with others may influence the loci of control.

### 3.3 Control

pwMND wanted to be in control of the decisions they made about interventions; a preference that could be facilitated or threatened by interactions with others.

#### 3.3.1 Retaining autonomy over decisions

pwMND consistently reported a preference to remain in control over decisions about interventions.^27^, ^29^, ^33^, ^37^, ^38^, ^39^, ^40^, ^51^ In line with pwMND preferences, HCPs and caregivers sought to protect pwMND right to self‐determination and empower pwMND to make their own decisions.^35^, ^38^, ^39^, ^46^

(P) ‘*John [dietician] came back out again and said as you get nearer for the PEG, don't let anybody bully you into it.’* ^38^

This quote points to the possibility that external agents may influence pwMND ability to exert autonomy during decision‐making, including convincing pwMND to make decisions that are not aligned with their preferences; a scenario that was described in a number of papers.^27^, ^32^, ^33^, ^37^, ^39^, ^40^, ^44^, ^46^, ^47^, ^50^ pwMND reported the perception of feeling pressured to accept interventions by HCPs was accentuated when approaches were repeated and from multiple different sources.^27^, ^30^, ^33^, ^40^, ^47^

(P) ‘*I mean the speech therapist came round, she said ‘well something you've got to start thinking about is this pipe’ and [MND Specialist Nurse] came round and she said about it and that other woman said something about it an' all. It felt like a lot of pressure was being put on me*.’^33^

In some studies, there was a sense of ‘side taking’, with HCPs and caregivers joining forces to project their own preferences for interventions.^39^, ^48^ Some HCPs reported finding it challenging to present neutral information, feeling a responsibility to advocate for the timely uptake of interventions.^39^

#### 3.3.2 When the perception of choice runs out

pwMND perception of having choice about interventions was narrowed or removed by HCPs limiting the options presented or the significance of the health threat presented by the disease. While HCPs reported presenting options to commence or decline interventions, there were also examples where HCPs withheld options^34^, ^39^, ^50^ or framed alternatives in a way to make them not even appear to be a choice.^44^, ^48^

(H) ‘*I haven't discussed noninvasive ventilation because…how would he ever cope? So, I've made, on best interests, not to start discussing those issues…. His anxiety, it's just going to raise his anxieties.’* ^39^

pwMND were able to enact agency over decisions while they were coping with symptoms and choosing to delay or decline interventions. However, when symptoms presented a significant threat to pwMND health or life, interventions were often perceived to be the only choice available to prevent serious consequences.^32^, ^33^, ^34^, ^37^, ^39^, ^42^, ^46^, ^48^

#### 3.3.3 Planning for the ‘future self’

Decision‐making often involved pwMND attempting to visualise how their future self may value an intervention. pwMND reported struggling to imagine how they would feel about an intervention when living with increased disease burden.^37^, ^43^, ^46^, ^49^ Acceptance of disease progression was associated with pwMND planning ahead through either commencing interventions before them being needed or making advanced decisions to decline interventions altogether.^28^, ^29^, ^33^, ^36^, ^37^, ^39^, ^40^, ^41^, ^46^ Planning ahead could allow pwMND to remain in control of their destiny, including those at risk of cognitive decline.^25^, ^35^, ^39^, ^51^ However, other pwMND feared that interventions would be commenced or continued despite them experiencing a poor quality of life.^30^, ^39^, ^50^, ^51^

pwMND wanted to remain in control of decision‐making. Interactions with HCPs and caregivers influenced pwMND perception choice and agency over their decisions. The timing of interventions is a challenging issue for all involved.

### 3.4 Tipping the balance

The timing of initiating discussions and commencing interventions was a source of conflict for all stakeholders. Commencing interventions often required pwMND to reach a subjective tipping point informed by disease progression, acceptance of need and recommendations of HCPs.

#### 3.4.1 Readiness to start the conversation

While HCPs advocated for an individualised patient‐centred approach, HCPs supported introducing intervention options early during the disease course.^17^, ^39^ In addition to referring to signs of disease progression, HCPs reported making subjective assessments of pwMND psychological readiness to discuss interventions.^39^, ^45^ Some pwMND reached crisis point without having the opportunity to understand their options.^34^ While early discussions about interventions allowed more time to consider the options,^33^, ^38^, ^39^, ^43^, ^46^ for some pwMND having these discussions conflicted with a personal preference for focusing on the present day challenges of the disease.^29^, ^33^, ^37^, ^38^, ^42^, ^43^, ^46^

(P) ‘*No, no, no. I'd rather not know until there's a reason to know. I don't want to know all the nasty possibilities that might be in front of me. You know, I just don't want to know the detail, until there's a reason to know it*.’^33^

#### 3.4.2 Reaching the tipping point

With regard to the timing of interventions, HCP preferences for ‘earlier rather than later’ were informed by previous clinical experience of poor outcomes associated with late intervention.^33^, ^36^, ^39^, ^42^, ^43^, ^46^, ^49^ pwMND perception of intervention need can deviate from that of HCPs, preferring to continue to cope with symptoms rather than introduce an intervention into their lives.^27^, ^29^, ^33^, ^45^ The tipping point was defined by acceptance of disease progression, experience of adverse consequences related to the health threat or recognition that the benefits of an intervention now outweigh the risks.^25^, ^32^, ^33^, ^34^, ^36^, ^37^, ^38^, ^39^, ^43^, ^46^, ^48^, ^49^

(P) ‘*It was a gradual change in my decision. As swallowing got worse I couldn't drink water and thickened stuff was not pleasant. So I thought to be hydrated in summer was a good idea and also taking medications would be easier through the tube.’* ^36^

pwMND who perceived that their symptoms were not causing significant problems could continue to defer decisions about interventions, even when presented with objective measures of functional decline or the direct observations of others.^27^, ^39^, ^42^ The uncertain rate of disease progression made it difficult for pwMND to identify the ‘right time’ to start an intervention and could limit the time available to make decisions especially when there was a serious health threat.^32^, ^34^, ^39^, ^42^, ^45^, ^46^, ^48^

(C) ‘*You don't know how it's going to proceed…whether it's going to proceed quickly…the uncertainty makes it far more difficult to make decisions because you don't know what tomorrow's going to bring. I mean I'm sure [patient] would find it easier, if she knew how it was going to progress and if we had some idea of timescale.’* ^46^

HCP guidance and recommendations were reported to influence the timing of interventions.^33^, ^36^, ^38^, ^39^, ^42^, ^46^ Some pwMND found information about timing of interventions inadequate and expected HCPs to use their experience to provide more concrete guidance.^33^, ^46^ However, as described earlier, the approach taken by HCPs when revisiting decisions about interventions can result in pwMND feeling pressurised to commence interventions or change previous decisions.

(C) ‘*They say make sure it's done sooner rather than later but what is sooner rather than later? They don't say we're talking next month, no definite time, it's down to you*.’^33^

## 4 DISCUSSION

### 4.1 Summary of findings

The review findings describe how decisions about interventions generate an emotional response that extends beyond consideration of the functional issues and are the consequence of interactions with a range of external agents. pwMND reflect on how life with an intervention will alter their independence, freedom and survival compared to continuing with the status quo, including concern for the impact on their caregivers. HCPs and caregivers played an integral role during decision‐making, through supporting pwMND to understand their options, retain control and make decisions aligned with their values. The sense of choice and agency experienced by pwMND during decision‐making was mediated by disease progression and the actions of others. The timing of interventions is a source of uncertainty, with decisions being driven by symptom severity, HCP guidance and pwMND acceptance of need for intervention.

### 4.2 The emotional response to interventions

Most studies reported pwMND associating interventions with a life with increased restrictions, burden and reliance on others. These findings are consistent with Foley et al.^58^ exploration of the ‘meaning of quality of life’ for pwMND, where participants reported an emotional response to the losses experienced due to the disease and fought to maintain normality and their own identity. Discussions about interventions signified a step‐change in disease progression requiring pwMND to undertake existential deliberations about the value of artificially prolonging their life. Though pwMND report prolonging life to be a driver for accepting interventions, many base decisions on the acceptability of the predicted quality of that life, reflecting on how interventions will impact on their lives and those of their caregivers in the present and the future. Aligned with previous research and theory of human behaviour, emotional responses (e.g., fear and anxiety) to the implications of an intervention may result in disengagement from decision‐making discussions or deferring the making of a decision.^18^, ^59^ These findings highlight the importance of actively exploring pwMND representations of interventions. Engagement in decision‐making discussions may be facilitated through psychological interventions that identify and address the emotional response to interventions.^59^, ^60^, ^61^, ^62^

### 4.3 Intervention timing

Following the decision about ‘if’ they should accept intervention, pwMND are faced with the uncertainty about ‘when’ to commence the intervention. The review findings describe how decisions about timing of interventions are influenced by pwMND acceptance or understanding of need, and their response to HCP guidance. Decisions about the timing of interventions are important, with delays being associated with poorer outcomes, particularly in relation to gastrostomy placement.^11^, ^63^, ^64^, ^65^ Such studies underpin the rationale for discussing and commencing interventions earlier in the disease course.^7^, ^8^, ^9^, ^66^ However, pwMND decisions are not informed solely by deteriorating clinical markers but also how aligned outcomes of an intervention are with pwMND values and priorities including the perceived impact on caregivers.

The perceptions of the disease and interventions are informed by an iterative updating of illness cognitions in response to pwMND evolving acceptance and adaptation to the relentless changes in their condition.^16^, ^61^, ^67^, ^68^ The review confirms that pwMND need support to understand how they will perceive living with the increased symptom burden associated with disease progression. Such support may enable pwMND to accept the need for interventions earlier or, conversely, facilitate informed decisions about delaying or declining interventions.

### 4.4 Autonomy and control

The review captured how external agents can facilitate individual autonomy,^69^, ^70^ an ethical principle valued by pwMND, through protecting the right to self‐determination and provision of information. Caregivers play a multilayered role during decision‐making. Consistent with a critique of autonomy in end‐of‐life care, pwMND made decisions in collaboration with and through concern for their caregivers.^71^ When pwMND decisions are based on concern for caregiver burden or prioritisation of caregiver preferences, an individualistic conceptualisation of autonomy could be perceived to be threatened. The social and interactional context in which pwMND make decisions in collaboration with caregivers may be better aligned with a relational conceptualisation of autonomy and is consistent with the principles of shared decision‐making.^71^, ^72^ However, a line is crossed when dialogue with external agents disrupts the patient's preference to make decisions based on their own values.^72^ This was evident in the review with pwMND sometimes feeling pressurised to accept interventions by caregivers and HCPs.

HCPs have an ethical and professional obligation to support pwMND to make autonomous, informed, values‐based decisions about interventions.^69^, ^73^, ^74^ The contrasting preferences of pwMND for, and emotional responses to, disease and intervention‐related information present a challenge for HCPs attempting to balance the ethical principles of beneficence and autonomy.^69^, ^75^ Despite being motivated by a responsibility to promote positive health outcomes, multiple HCPs repeatedly revisiting discussions placed some pwMND under pressure to accept interventions. Conversely, pwMND can expect HCPs to use their expertise to provide direction about if, and when, to start an intervention. While taking what may be considered a more paternalistic approach risks paying less attention to pwMND values,^76^ HCP recommendations can support patients to navigate the uncertainty present during decision‐making.^77^, ^78^, ^79^ HCPs could positively contribute to relational autonomy by ensuring recommendations are grounded in a knowledge of pwMND preferences, goals and values.^75^

Consistent with a previous review^21^ only one study^35^ referred to the impact cognitive impairment may have on pwMND decision‐making. Cognitive impairment, which is common in MND,^80^, ^81^ has been identified as a barrier to autonomous decision‐making,^19^ and associated with reduced acceptance^82^ or delayed starting^17^ of interventions. Further research is required to explore how pwMND with mild‐moderate cognitive deficits can be supported to engage in decision‐making, and elicit pwMND preferences (i.e., advanced decisions) in the context of fluctuating and deteriorating mental capacity to make their own decisions.

### 4.5 Strengths and limitations

A limitation of this review was the absence of a second reviewer to shortlist the references for inclusion, extract data and independently code the data which may limit the credibility of the study. However, this is mitigated to a degree by some of the methodological strengths of the review such as the systematic approach to the study design, including a comprehensive bibliographic and supplementary search strategy, a second reviewer screening 10% of references at the abstract and full‐text stages and discussions with supervisory team when developing the analytical themes.

A further limitation relates to the review not making any distinctions between decisions made about gastrostomy, non‐invasive ventilation and invasive ventilation. These are interventions, with differing clinical indications, outcomes and implications for pwMND and their caregivers. There may have been valuable insights gained from exploring the consistencies and differences of the decision‐making process for the different interventions. A review of the data did not reveal enough rich data to provide a credible account of any such differences. Future qualitative research may benefit from comparing the contextual factors that influence decisions about different interventions to generate deeper analytical insights.

### 4.6 Reflexivity

S.W. is a dietitian with experience of supporting pwMND to make decisions about gastrostomy placement. Prior experience and opinions of a researcher have the potential to affect how data are analysed and interpreted.^83^ Any such influence was mediated through staying close to the primary studies, maintaining a clear audit trail of the analysis process, and regular discussions with the research team about the decisions made during the screening process and analysis.

### 4.7 Implications for practice

The emotional response to interventions highlights the importance of exploring the value pwMND place on the implications and outcomes of an intervention beyond the concern for clinical outcomes. Decision‐making should address the issues that matter most to pwMND including the existential debate pwMND have about an intervention's potential to affect their quality and length of life. While HCPs have an ethical responsibility to communicate with pwMND about the predicted disease course and timely consideration of interventions caution should be taken to respect individuals' preferences for information and control. Acknowledging early in the decision‐making process, the contribution pwMND, caregivers and HCPs make towards a relational conceptualisation of autonomy may facilitate a shared understanding of each other's role and boundaries, and promote meaningful engagement in decision‐making discussions.^71^

## 5 CONCLUSION

Addressing the emotional response pwMND have to the prospect of interventions could improve engagement in decision‐making discussions. HCPs and caregivers have a complex ethical path to navigate when seeking to protect pwMND autonomy while simultaneously attempting to optimise health outcomes. Decision support strategies that mediate the emotional response and promote autonomy have the potential to enable pwMND to make timely, values‐based decisions about interventions.

## AUTHOR CONTRIBUTIONS

*Funding acquisition*: Sean White, Alicia O'Cathain, Vanessa Halliday and Christopher McDermott*. Conceptualisation and design*: Sean White, Alicia O'Cathain, Vanessa Halliday and Christopher McDermott. *Study screening and selection*: Sean White and Liz Croot. *Analysis, synthesis and interpretation*: Sean White, Alicia O'Cathain, Vanessa Halliday, Liz Croot and Christopher McDermott. *Writing—original draft*: Sean White. *Writing—review and editing*: Sean White, Alicia O'Cathain, Vanessa Halliday, Liz Croot and Christopher McDermott. *Supervision*: Alicia O'Cathain. Vanessa Halliday and Christopher McDermott have provided on‐going supervision of the work.

## CONFLICT OF INTEREST STATEMENT

The authors declare no conflict of interest.

## Supporting information

Supporting information.

Supporting information.

Supporting information.

## Data Availability

No primary data were generated, analysed or stored as part of this study.
