# Supplementary material for: Factors influencing decisions people with motor neuron disease make about gastrostomy placement and ventilation: A qualitative evidence synthesis
Source: Health Expect. 2023 May 31;26(4):1418–35. doi: 10.1111/hex.13786 (PMC10349261; doi:10.1111/hex.13786)
Supplement: Supplementary file 1 — Supporting information. [file HEX-26--s002.docx]

**APPENDIX A**

See table A1

Table A1. A summary of how the review met the recommendations of the Enhancing transparency in reporting the synthesis of qualitative research (ENTREC) statement^24^

| Item | Guide and description |
| --- | --- |
| Aim | Aim of the review is clearly described. |
| Synthesis methodology | Rationale given for thematic synthesis |
| Approach to searching | A comprehensive search strategy was planned to seek all available studies that relate to the research question. |
| Inclusion criteria | Inclusion criteria stated including population, language, year limits, type of publication, and study type. |
| Data sources | All database and supplementary sources included with dates of searches. |
| Electronic Search strategy | Search strategy described including the databases searched and the scope of the supplementary searches. Example of the full Medline search terms used included in appendix. |
| Study screening methods | Comprehensive description of study screening method, including role of second reviewer who screened 10% of the title/abstracts and full texts. |
| Study characteristics | Description and table of study characteristics including year of publication, country, population, number of participants, data collection, methodology, aim. |
| Study selection results | Study selection results provided in text and PRISMA flow diagram including data sources, duplicates and reasons for exclusions. |
| Rationale for appraisal | The results of the quality appraisal using the CASP qualitative appraisal tool was presented to inform the readers critical appraisal of the review findings. |
| Appraisal items | CASP qualitative appraisal tool used to assess the quality of the included studies |
| Appraisal process | Appraisal process conducted by single reviewer |
| Appraisal results | Appraisal results present in tabular form and included as an appendix. |
| Data extraction | All the PDFs of the selected studies were imported into NVivo for data extraction. Only text in the findings/results sections of publications were analysed. |
| Software | EndNote used to store the study publications. Excel used to manage the study selection process. NVivo used to perform the line by line coding and to manage the data while developing the descriptive and analytical themes. |
| Number of reviewers | The primary investigator (SW) screened all title/abstracts, full texts, completed quality assessment and analysis and synthesis. Second reviewer (LC) screened 10% of title/abstracts and full texts. |
| Coding | Thematic synthesis including line by line coding described |
| Study comparison | Where present differing perspectives on similar concepts were highlighted and discussed. The reviewer actively looked for alternative explanations for concepts throughout the analysis phase. |
| Derivation of themes | An inductive approach to the synthesis was taken |
| Quotations | Mostly participant quotes were included, including indicating whether the quotes were from a pwMND (P), caregiver (C), or HCP (H). A few author extracts (R) were also included within the findings. |
| Synthesis output | A pathway of care model was developed to capture |
