# Supplementary material for: Factors influencing decisions people with motor neuron disease make about gastrostomy placement and ventilation: A qualitative evidence synthesis
Source: Health Expect. 2023 May 31;26(4):1418–35. doi: 10.1111/hex.13786 (PMC10349261; doi:10.1111/hex.13786)
Supplement: Supplementary file 2 — Supporting information. [file HEX-26--s001.docx]

**APPENDIX B**

See below for the full Medline on Ovid search strategy run on 27/9/2021.

Ovid MEDLINE(R) and Epub Ahead of Print, In-Process, In-Data-Review & Other

Non-Indexed Citations, Daily and Versions(R) <1946 to September 27, 2021>

1 motor neuron disease*.ti,ab.

2 motor neurone disease*.ti,ab.

3 MND.ti,ab.

4 exp Motor Neuron Disease/

5 Amyotrophic lateral sclerosis.ti,ab.

6 ALS.ti,ab.

7 exp Amyotrophic Lateral Sclerosis/

8 Lou Gehrig* disease*.ti,ab.

9 Progressive bulbar pals*.ti,ab.

10 exp bulbar palsy, progressive/

11 Progressive muscular atroph*.ti,ab.

12 Primary lateral scleros*.ti,ab.

13 1 or 2 or 3 or 4 or 5 or 6 or 7 or 8 or 9 or 10 or 11 or 12

14 decision.ti,ab.

15 exp Decision-making/

16 (mak* adj2 decision*).ti,ab.

17 decision-making.ti,ab.

18 (shared adj2 decision).mp. [mp=title, abstract, original title, name of substance

word, subject heading word, floating sub-heading word, keyword heading word,

organism supplementary concept word, protocol supplementary concept word, rare

disease supplementary concept word, unique identifier, synonyms]

19 patient engage*.ti,ab.

20 exp Patient Participation/

21 patient participat*.ti,ab.

22 exp Decision-making, Shared/

23 exp Patient Preference/

24 choice*.ti,ab.

25 option*.ti,ab.

26 preference*.ti,ab.

27 advance* care plan*.ti,ab.

28 advance* decision*.ti,ab.

29 exp advance care planning/

30 14 or 15 or 16 or 17 or 18 or 19 or 20 or 21 or 22 or 23 or 24 or 25 or 26 or 27

or 28 or 29

31 exp Qualitative Research/

32 qualitativ*.ti,ab.

33 interview*.ti,ab.

34 exp interview/

35 focus group*.ti,ab.

36 exp focus groups/

37 thematic analys*.ti,ab.

38 conversational analys*.ti,ab.

39 content analys*.ti,ab.

40 phenomenol*.ti,ab.

41 ethnograph*.ti,ab.

42 exp Anthropology, Cultural/

43 attitude*.ti,ab.

44 experience*.ti,ab.

45 perception*.ti,ab.

46 exp Attitude to Health/ or exp Perception/

47 theme*.ti,ab.

48 (grounded adj (theor* or stud* or research or analys?s)).mp. [mp=title,

abstract, original title, name of substance word, subject heading word, floating subheading word, keyword heading word, organism supplementary concept word,

protocol supplementary concept word, rare disease supplementary concept word,

unique identifier, synonyms]

49 (biographical adj1 method).ti,ab.

50 theoretical sampl*.ti,ab.

51 (purpos* adj4 sampl*).ti,ab.

52 (observational adj1 method*).ti,ab.

53 (constant adj (comparative or comparison)).ti,ab.

54 ((discourse* or discurs*) adj3 analys?s).ti,ab.

55 narrative analys*.ti,ab.

56 (social construct* or post-modern* or postmodern* or post-structural* or

poststructural* or post structural* or post modern* or feminis*).mp. or interpret*.ti,ab.

[mp=title, abstract, original title, name of substance word, subject heading word,

floating sub-heading word, keyword heading word, organism supplementary concept

word, protocol supplementary concept word, rare disease supplementary concept

word, unique identifier, synonyms]

57 31 or 32 or 33 or 34 or 35 or 36 or 37 or 38 or 39 or 40 or 41 or 42 or 43 or 44

or 45 or 46 or 47 or 48 or 49 or 50 or 51 or 52 or 53 or 54 or 55 or 56

58 exp Gastrostomy/

59 gastrostom*.ti,ab.

60 PEG.ti,ab.

61 RIG.ti,ab.

62 percutaneous endoscopic gastrostom*.ti,ab.

63 Percutaneous image guided gastrostom*.ti,ab.

64 per-oral image guided gastrostom*.ti,ab.

65 radiologically inserted gastrostom*.ti,ab.

66 percutaneous radiological gastrostom*.ti,ab.

67 g-tube.ti,ab.

68 feed* tube.ti,ab.

69 tube feed*.ti,ab.

70 artificial feed*.ti,ab.

71 artificial nutrition.ti,ab.

72 enteral feed*.ti,ab.

73 enteral nutrition.ti,ab.

74 exp enteral nutrition/

75 non-oral feed*.ti,ab.

76 exp Ventilation/ or exp Noninvasive Ventilation/

77 non-invasive ventilation.ti,ab.

78 noninvasive ventilation.ti,ab.

79 non invasive ventilation.ti,ab.

80 NIV.ti,ab.

81 exp Respiration, Artificial/

82 mechanical ventilation.ti,ab.

83 tracheostomy.ti,ab.

84 life prolonging treatment.ti,ab.

85 life prolonging intervention.ti,ab.

86 58 or 59 or 60 or 61 or 62 or 63 or 64 or 65 or 66 or 67 or 68 or 69 or 70 or 71

or 72 or 73 or 74 or 75 or 76 or 77 or 78 or 79 or 80 or 81 or 82 or 83 or 84 or 85

87 30 or 86

88 13 and 57 and 87
