# Supplementary material for: Factors influencing decisions people with motor neuron disease make about gastrostomy placement and ventilation: A qualitative evidence synthesis
Source: Health Expect. 2023 May 31;26(4):1418–35. doi: 10.1111/hex.13786 (PMC10349261; doi:10.1111/hex.13786)
Supplement: Supplementary file 3 — Supporting information. [file HEX-26--s003.docx]

**APPENDIX C**

See table C1

Table C1. Summary of the supplementary search strategy.

| Supplementary search strategy | Searches performed |
| --- | --- |
| Thesis search | Search of the ProQuest dissertation and theses database |
| Reference list | Reference lists of all publications included in the review and 23 relevant reviews |
| Forward citation search | Web of Science forward citation search of all publications included in the review on 25/10/21 |
| Journal contents | Scanned the previous 3 years online journal contents of ‘Amyotrophic Lateral sclerosis & Frontotemporal Degeneration’ on 14/10/21 |
| Conference proceedings | Search the previous 3 years (2018, 2019 and 2020) abstracts for the annual International Symposium on ALS/MND checked on 8/10/21. |
| Author and subject matter experts | The authors from the publications included in the review and selected academic and healthcare professional subject matter experts were emailed to identify any references that the searches may have missed (9 replied). The MND Association and BDA neurology specialist group were also contacted |
